# Hazard Prevention Regarding Occupational Accidents Involving Blue-Collar Foreign Workers: A Perspective of Taiwanese Manpower Agencies

**DOI:** 10.3390/ijerph13070706

**Published:** 2016-07-13

**Authors:** Huan-Cheng Chang, Mei-Chin Wang, Hung-Chang Liao, Shu-Fang Cheng, Ya-huei Wang

**Affiliations:** 1 Division of Nephrology, Department of Medicine, Landseed Hospital, No. 77, Guangtai Rd., Pingzhen City, Taoyuan 324, Taiwan; changhc@landseed.com.tw; 2 Department of Health Care Management, Chang Gung University, No. 259, Wenhua 1st Rd., Guishan Dist., Taoyuan 33302, Taiwan; 3 Noble Health Management Center, Landseed Hospital, No. 77, Guangtai Rd., Pingzhen City, Taoyuan 324, Taiwan; wangmeria@landseed.com.tw; 4 Department of Health Services Administration, Chung Shan Medical University, No. 110, Sec. 1, Jian-Koa N. Rd., Taichung 402, Taiwan; 5 Department of Medical Management, Chung Shan Medical University Hospital, No. 110, Sec. 1, Jian-Koa N. Rd., Taichung 402, Taiwan; yhuei@csmu.edu.tw; 6 Institute of Labor, Occupational Safety and Health, Ministry of Labor, Executive Yuan, No. 99, Lane 407, Hengke Rd., Sijhih District, New Taipei City 22143, Taiwan; chengsf@mail.ilosh.gov.tw; 7 Department of Applied Foreign Languages, Chung Shan Medical University, No. 110, Sec. 1, Jian-Koa N. Rd., Taichung 402, Taiwan

**Keywords:** occupational accidents, blue-collar foreign workers, blue-collar foreign workers’ agencies

## Abstract

Since 1989, blue-collar foreign workers have been permitted to work in Taiwanese industries. Most blue-collar foreign workers apply for jobs in Taiwan through blue-collar foreign workers’ agencies. Because blue-collar foreign workers are not familiar with the language and culture in Taiwan, in occupational accident education and hazard prevention, the agencies play an important role in the coordination and translation between employees and blue-collar foreign workers. The purpose of this study is to establish the agencies’ role in the occupational accidents education and hazard prevention for blue-collar foreign workers in Taiwan. This study uses a qualitative method—grounded theory—to collect, code, and analyze the data in order to understand the agencies’ role in occupational accident education and hazard prevention for blue-collar foreign workers in Taiwan. The results show that the duty of agencies in occupational accident education and hazard prevention includes selecting appropriate blue-collar foreign workers, communicating between employees and blue-collar foreign workers, collecting occupational safety and health information, assisting in the training of occupational safety and health, and helping blue-collar foreign workers adapt to their lives in Taiwan. Finally, this study suggests seven important points and discusses the implementation process necessary to improve governmental policies. The government and employees should pay attention to the education/training of occupational safety and health for blue-collar foreign workers to eliminate unsafe behavior in order to protect the lives of blue-collar foreign workers.

## 1. Introduction

Many studies show that, compared to their local counterparts, migrant workers face greater challenges in adapting to their overall living environments, in addition to higher risks of occupational accidents and injuries. A French study [1] stated that the nature of tasks and employment (e.g., short-term or part-time employment) makes severe occupational risks more likely for migrant workers. As mentioned in the same study, thirty percent of the workers in France are non-French, an excessively large percentage of migrant workers in France were accident victims, and industrial accidents caused more than thirty percent of lifelong disabilities of workers nationwide. Spanish survey results indicate the country’s 2004 incidence rate of occupational accidents was 53% for all workers and 73% for migrant workers on temporary contracts. Regarding the occupational distribution of workers, under-qualification accounted for 31% of 2004 Spanish occupational accidents involving migrant workers, a group often assigned dangerous tasks, and 20% of total accidents [1]. Based on the above statistics, some scholars [2] have pointed out the alarming risks and accidents facing a majority of migrant workers, who perform the most dangerous, unhealthy, and unskilled tasks. The jobs of those who lack language skills or are underprivileged are even riskier, according to research [3].

Some reports have concluded that migrant workers have a greater chance of suffering from hearing and musculoskeletal disorders, perhaps because of their lack of professional competencies. As a rule, migrant workers are unlikely to be assigned knowledge-oriented tasks, and most of them are engaged in manual labor instead. With little occupational training, they are almost always forced to work jobs in noisy environments that require sheer physical strength. These jobs explain why appropriate and accessible occupational medical services are critical for the health of migrant workers [4]. Article 7 of the International Covenant on Economic, Social and Cultural Rights [5] says:

“*The States Parties to the present Covenant recognize the right of everyone to the enjoyment of just and favorable conditions of work which ensure, in particular… fair wages and equal remuneration for work of equal value without distinction of any kind, in particular women being guaranteed conditions of work not inferior to those enjoyed by men, with equal pay for equal work…safe and healthy working conditions… rest, leisure and reasonable limitation of working hours and periodic holidays with pay, as well as remuneration for public holidays.*”

Efforts are needed to ensure that all laborers, regardless of nationality, are employed in environments with job equality, safety, and health. Blue-collar foreign workers, among others, often leave their families behind for jobs to which they must accustom themselves, mentally and/or physically. In totally unfamiliar living environments, cultural and geographical differences become sources of stress and indirect causes of occupational safety problems, which is why occupational safety and health (OSH) risks facing blue-collar foreign workers deserve extra attention from governments worldwide and also from the businesses that hire such workers. Most blue-collar foreign workers in Taiwan are recruited through manpower recruitment and placement service providers (hereinafter referred to as “manpower agencies”). Blue-collar foreign workers unfamiliar with the Taiwanese language, and businesses that hire these workers, rely on these manpower agencies for labor management and translation services.

In Taiwan, the number of blue-collar workers has reached 551,596 workers on 31 December 2014. Laborers’ OSH education/training is an integral part of efforts to reduce occupational accidents and also an issue of pressing urgency. Taiwan’s Occupational Safety and Health Act [6] requires that employers offer employees OSH education and training programs as required by the employees’ specific tasks and for disaster prevention purposes. In other words, laborers are supposed to receive OSH training and learn to safely perform their tasks while preventing disasters. 

The primary purpose of this study is to clarify the role, and identify the flaws, of manpower agencies in OSH education/training and hazard prevention efforts targeting blue-collar foreign workers, and present recommendations for all parties involved in those efforts, in particular the manpower agencies and the businesses that hire blue-collar foreign workers. This study also discusses ways to improve the implementation of governmental policies.

## 2. Methodology

### 2.1. Determining the Research Process and Sampling

This study was conducted in two phases in order to examine the role of manpower agencies in preventing occupational accidents. Firstly, the manpower agencies of two selected businesses were contacted for interviews with their contract factory interpreters, so as to gain insight into how those interpreters contribute to the OSH education/training for blue-collar foreign workers. One of the businesses hired 50 blue-collar foreign workers and the other 300; the contract factory interpreters for both businesses had worked in their positions for more than five years. After selecting the businesses, focused interviews of manpower agencies occurred in three regions across Taiwan (Taipei, Taichung, and Kaohsiung). All the manpower agencies interviewed are primarily importers of factory workers and listed by the Bureau of Employment and Vocational Training (BEVT) as well-performers in a nationwide evaluation. Interview questions in the passage below were asked to better understand the manpower agencies’ active role in preventing occupational accidents throughout a blue-collar foreign worker’s employment in Taiwan, from the moment of the work application to the worker’s departure from Taiwan after the contract expired.

The following in-depth interview questions were posed to contract factory interpreters:

(1) What is your greatest challenge concerning the OSH education/training programs for blue-collar foreign workers?
(2) What do you think is the best approach to OSH education/training for blue-collar foreign workers?
(3) Can you think of any other way to make blue-collar foreign workers more aware of OSH?

Major questions for focused interviews were the following:

(1) What kind of assistance is available for an employer to enhance OSH education/training for blue-collar foreign workers?
(2) What kind of assistance is available for an employer to raise its blue-collar foreign workers’ alertness against occupational injuries?
(3) From your experiences, what are the flaws in, or hindrances to, employers’ efforts to implement blue-collar foreign workers’ OSH education/training programs? What kind of assistance is available to remedy those flaws or hindrances?

### 2.2. Data Analysis

On the basis of the *grounded theory*, an inductive analysis was conducted qualitatively in this study, in an attempt to look at the topic of interest from the viewpoint of the person(s) to be studied, or subject(s) [7]. The researcher derived transcripts from audio recordings to extract/construct data, as well as theoretical elements, in a model of grounded theory. This grounded theory-based study follows a process that involves the selection of interviewees, interviews and production of interview transcripts, theoretical sampling, comparative and open coding, selective coding, the development of a multiphase model for the theory, and a presentation of the conclusion.

#### 2.2.1. Theoretical Sampling

Theoretical sampling was used here for data collection and analysis. In this study, the participants were selected based on the backgrounds in order to obtain diverse perspectives on this issue. Also, to respect the participants’ dignity and protect their confidentiality, in the study, the participants’ identities were disconnected. Table 1 shows the participants’ genders, educational levels, experience levels, contract factories, and the number of blue-color workers with whom they have dealt. At the stage of theoretical sampling, explicit phenomena/incidents mentioned in the transcripts were examined, with the important phenomena extracted as “key points” for coding [7]. For example, an excerpt of the transcript says: “*Manpower agencies are an intermediary institution between a business and the blue-collar foreign workers it already hires or plans to hire. Based on the nature of job openings at the hiring business (A-1), a manpower agency selects the right blue-collar foreign workers (A-2).*” Table 2 shows theoretical sampling as compared to comparative and open coding. The process of data collection and analysis continued until the point of theoretical saturation was reached, that is, the point at which no new data or relationships emerged from the interviews and analysis [7]. 

#### 2.2.2. Comparative and Open Coding

At this stage, phenomena/incidents revealed in theoretical sampling were compared for contextual meanings, and key points similar in meaning were grouped together. For instance, an excerpt of the transcript says: “*During the education/training sessions, some businesses have the new recruits advised by senior laborers (E-1) while some hire manpower agencies to offer round-the-clock, in-plant services (E-2)*.” These key points fall under the *in-plant services* category. Table 1 shows the details of comparative and open coding.

#### 2.2.3. Selective Coding

At the stage of selective coding, concepts of higher levels or greater importance (i.e., core concepts) were induced from each category according to the research topic [7]. For example, categories such as “Sources of OSH teaching materials”, “In-plant services”, and “Interpreting services as part of OSH training programs” pertain to how the manpower agencies support OSH education/training efforts and therefore fall under Concept D: Assistance in OSH education/training for blue-collar foreign workers. Table 3 shows details of selective coding. 

### 2.3. Constructing the Theory

The theoretical foundation of this study was constructed on the basis of concept categories stemming from selective coding. Judging from the interview results, the researchers integrated the outcome of theoretical sampling to build a theory that describes the five aspects of manpower agencies’ role in occupational safety, as stated below:

1. An intermediary institution that selects blue-collar foreign workers for the employers:

Manpower agencies act as intermediary institutions between a business and the blue-collar foreign workers it already hired or plans to hire. According to the nature of job openings at the hiring business (A-1), the agencies select the right blue-collar foreign workers (A-2). Although Taiwanese manpower agencies seek the help of their foreign-based counterparts in selecting workers overseas (A-3), some candidates selected/recommended by the latter fail to meet the hiring businesses’ needs (A-4). It is, therefore, imperative that Taiwanese manpower agencies carefully control the quality of imported human resources during the interviews (A-5) and reduce the risk of occupational hazards involving incompetent blue-collar foreign workers.

2. A bridge of communication between blue-collar foreign workers and employers:

Most businesses hiring blue-collar foreign workers have no more Chinese-language requirements for them than the basic communication skills (B-1). This lax requirement explains why such workers obviously are lacking in Chinese-language skills upon arriving in Taiwan, and also why the manpower agencies must act as a bridge of communication between blue-collar foreign workers and their employers. To a certain extent, workers hailing from English-speaking countries display a satisfactory level of English proficiency. Taiwanese businesses that adopt English as a primary language of communication or interview new recruits in English through manpower agencies overseas (B-2) are more likely to select competent employees, even though such businesses represent a small percentage of all Taiwanese employers. The truth is, language barriers are common among most Taiwanese businesses seeking blue-collar foreign workers (B-3). Moreover, the fact that a majority of blue-collar foreign workers come from non-English-speaking countries widens the gap between the employees and employers in terms of education/training and work-related instructions. The competencies of interpreters hired by manpower agencies, which are responsible for tackling the aforementioned gap, help bolster the blue-collar foreign workers’ adaptability to an unfamiliar environment while reducing language barriers.

3. Gathering information for OSH education/training:

Most blue-collar foreign workers receive in their home countries training from manpower agencies in occupational skills, but not OSH knowledge (C-1). It is even possible that some such workers never received any training at all (C-2). When a blue-collar foreign worker arrives in Taiwan, the manpower agency will help the employer offer education/training programs pertaining to both job performance and OSH. Because potential occupational accidents vary among businesses, manpower agencies, as “co-trainers” with the hiring businesses, are expected to discuss with the latter the potential occupational accidents linked to the job openings, their causes, and the other important information before recruiting (C-3), in order to expedite the preparation of education/training materials. The authorities may also compile a standard version of industry-specific OSH education/training materials with a focus on the highly probable accidents and incorporate the required occupational skills into those OSH materials [8]. That way, the manpower agencies can follow a set of well-defined rules, knowing how to prepare for, implement, and promote an OSH education/training program.

4. Assistance in OSH education/training for blue-collar foreign workers:

(a) Sources of OSH teaching materials

Every newly recruited blue-collar foreign worker is required to receive OSH education/training from the hiring business immediately after coming on board (D-1). Generally speaking, OSH education/training materials may be compiled by the hiring businesses, provided by the CLA, or edited by manpower agencies using information they have collected (D-2). Because the manpower agencies currently rely heavily on government information for such materials, the agencies may unwittingly provide blue-collar foreign workers with outdated information (D-3), leaving the OSH training ineffective. Therefore it is imperative that a manpower agency ensure the validity of training materials when supporting a hiring business’ OSH training efforts. 

(b) In-plant services

Although some hiring businesses give their new recruits access to senior workers’ guidance (E-1), some hire manpower agencies to offer round-the-clock, in-plant services (E-2). Such in-plant services, whether they are provided by senior laborers or manpower agencies, invariably take the form of on-site instructions offered in accordance with the nature of tasks (E-3). After all, only through on-site testing can the employer ensure that a blue-collar foreign worker really knows how to perform tasks correctly (E-4). The interpreter acts as an assistant/adviser during OSH education/training sessions (E-5) who answers questions or resolves the doubts of blue-collar foreign workers. With the help of interpreters, a business may add bilingual warning signs to the machinery and thus protect blue-collar foreign workers (E-6). 

(c) Interpreting services as part of the OSH training program

The manpower agencies’ interpreting services are performed mostly by foreign spouses of native Taiwanese citizens, in addition to a few students who grew up in the blue-collar foreign workers’ countries. The interpreters’ serious lack in OSH-relevant expertise (F-1) is attributed to the unavailable training at the manpower agencies, and most interpreters do nothing more than literally translating what the instructor says into English (F-2). Interpreters’ poor comprehension of OSH instructions may cause biased communication or even send the wrong message (F-3). Meanwhile, most businesses implement OSH training through oral instructions (F-4), which fails to give blue-collar foreign workers a realistic sense of potential occupational accidents (F-5). In fact, most workers do not know how serious a disaster or injury is until it occurs. Research shows that an unequivocal understanding of workplace threats is necessary to raise workers’ awareness of OSH, and videos showing images of real disaster scenes make it easier for blue-collar foreign workers to grasp the consequences of occupational accidents (F-6). When complemented by the interpreting service, such videos become powerful warnings that prevent biased communication. 

Another problem arising from the education/training process is that interpreters have poor comprehension of the instructions and may not understand how language usages differ among regions, including the workers’ home countries (F-7) and Taiwan. Because a Taiwanese technical term could mean something totally different in another language, an interpreting service rendered solely on the basis of Taiwanese employers’ terminology may cause misunderstandings, rather than conveying the intended messages (F-8), or even lead to accidents. Consequently, manpower agencies must require their interpreters to accomplish tasks with greater accuracy, regardless of the blue-collar foreign workers’ language skills (F-9). In other words, the interpreters must do their best to reduce such language barriers.

5. Helping blue-collar foreign workers adapt to life in Taiwan:

In addition to importing and training blue-collar foreign workers, Taiwanese authorities, employers, and the foreign workers themselves expect manpower agencies to help workers adapt to their Taiwanese jobs (G-1) and other aspects of life in Taiwan (G-2). It is inevitable that newly recruited blue-collar foreign workers feel overwhelmed by life in Taiwan, where everything seems unfamiliar and the jobs they are about to start plunge them into the unknown (G-3). Most of them suffer from tremendous mental, and subsequently physical, stress, in addition to the Taiwanese people’s prejudice against them. Research shows that a person working in a new environment will have problematic relationships with the environment and unbalanced physical/mental dynamics if he/she fails to meet the employer’s requirements or feels incompetent; in some cases, such people become runaways [9]. To avoid misunderstandings due to miscommunications (G-4) and/or cultural differences, manpower agencies should keep track of blue-collar foreign workers after they arrive in Taiwan and ensure their adaptability to the new environment (G-5). In other words, it is the manpower agencies’ responsibility to assess each blue-collar foreign worker’s competency in his/her position and serve as a bridge of communication between any incompetent worker and the hiring business. Taiwanese authorities, employers, and the foreign workers themselves also expect manpower agencies to advise blue-collar foreign workers on Taiwanese customs and ways of life. 

Manpower agencies exert a long-term, profound influence on how the blue-collar foreign workers they recruit adapt to their jobs and life in the labor-importing region [9]; they are responsible for helping blue-collar foreign workers adapt to all aspects of life in an unfamiliar region, reducing their day-to-day causes of stress, and ensuring workplace safety (G-6). If the hiring businesses with whom they partner are not cooperative in promoting OSH and pay no attention to such initiatives, the manpower agencies will have to rely on the government for effective supervision (G-7) for disaster prevention. 

## 3. Recommendations

Manpower agencies started to thrive in Taiwan decades ago, when the country decided to import a large number of blue-collar workers as a remedy for the biased labor force structure. Manpower agencies have many responsibilities: helping blue-collar foreign workers adapt to life and culture in Taiwan, assisting Taiwanese businesses in selecting blue-collar foreign workers, and giving occupational training to the selected laborers, etc. The problem is, Taiwanese manpower agencies make little effort to conduct OSH education/training for blue-collar foreign workers. 

Blue-collar foreign workers will be noticeably less aware of OSH than their Taiwanese counterparts if these workers do not learn enough about Taiwanese culture and language, especially because Taiwanese businesses give instructions entirely in the Chinese language. A national study covering 1982 to 2001 suggests that nearly 80% of the major occupational accidents in Taiwan were linked to the absence of OSH education/training programs, and the ratio of disaster victims untrained in OSH to those who were trained was 3.19 [10]. The findings indicate just how important and necessary OSH education/training is for blue-collar foreign workers. Special emphasis, therefore, should be attached to OSH education/training to protect blue-collar foreign workers from occupational safety risks stemming from language barriers, and also to give them a thorough understanding of workplace-relevant OSH information. 

Methodology-wise, this study is a qualitative attempt to examine opinions, problems, and recommendations about blue-collar foreign workers’ education/training efforts, as summarized below:

1. When seeking their foreign counterparts’ help in recruiting overseas, Taiwanese manpower agencies are supposed to specify the expected job skills, types of job openings, and workplace conditions in order to make sure only qualified people are hired. In other words, the manpower agencies are responsible for controlling human resources for the hiring businesses;
2. Because the types of occupational accidents vary among businesses, manpower agencies should obtain information from the hiring businesses regarding job openings (and, accordingly, the potential occupational accidents) before assisting foreign workers in OSH education/training, so as to ensure efficient preparation of the training materials;
3. Currently, most Taiwanese manpower agencies collect government information about OSH and use it as education/training materials. The authorities concerned, therefore, are advised to compile a standard version of industry-specific education/training materials with an emphasis on each industry’s potential accidents and required occupational skills or OSH knowledge. These standard versions of OSH training materials could serve as templates for local manpower agencies compiling their own materials;
4. As mentioned earlier, it is imperative that manpower agencies ensure the validity of government information used in their OSH training materials, so as to enhance the effects of OSH education/training programs as well as the trainees’ learning effects;
5. When giving on-site OSH instructions, Taiwanese authorities, employers, and the foreign workers themselves expect an interpreter to assist the trainees and answer their questions about how the machinery should be operated. With the help of interpreters, the businesses may also post warning signs in two languages near the machinery, in order to reduce occupational accidents caused by language barriers;
6. Currently, a majority of the interpreters appointed by Taiwanese manpower agencies are untrained in OSH and give literally translated OSH instructions to blue-collar foreign workers. Although the linguistic, terminological, and cultural differences among the three parties involved in OSH training (i.e., the instructors, interpreters, and blue-collar foreign workers) may cause misunderstandings throughout the course of communication, videos or images of what actually happened at disaster sites can raise the blue-collar foreign workers’ awareness of OSH and complement the manpower agencies’ interpreting services;
7. Manpower agencies should keep track of blue-collar foreign workers after they arrive in Taiwan for employment to ensure their adaptability to the new environment, and serve as a bridge of communication between the employer and any incompetent worker. They should also give advice to blue-collar foreign workers on how to relieve stress, both mentally and physically.

## 4. Implementing Improvements to Government Policies

Besides the above recommendations, the government can create policies to improve OSH, including the policy steps below:

1. Widen the variety of OSH teaching materials (spoken and written) in the native languages of blue-collar foreign workers.

Although government agencies are making an all-out effort to reduce workplace accidents and train blue-collar foreign workers in OSH, they also propose disseminating written and spoken teaching materials in foreign languages to the parties concerned. It is obvious that small and medium-sized Taiwanese enterprises do not spend enough time on developing, and indeed are not competent to develop, teaching materials that suit the nature of their businesses because they seldom have full-time employees who handle labor safety and health issues. A majority of small and medium-sized enterprises therefore expect the government to offer ready-made teaching materials for OSH training of blue-collar foreign workers in Taiwan. Given limited human, property, and financial resources, it is impossible for the government to provide every business with tailor-made, industry-specific teaching materials in the blue-collar foreign workers’ native languages. Consequently, the authorities concerned are advised to phase in a complete set of OSH teaching materials in other languages. The schedule for releasing these materials should be determined by how serious particular occupational accidents suffered by blue-collar foreign workers are, and how many such workers are hired in each industry.

2. Determine the qualifications for translators in blue-collar foreign workers’ OSH training programs.

According to Article 16 of Regulations for Occupational Safety and Health Education and Training [11], all employers shall give a newly hired or incumbent worker the required safety/health training before assigning him/her to a different job.

Currently, most OSH training programs for blue-collar foreign workers in Taiwan are conducted by qualified instructors in Mandarin, with on-site interpretation and assistance offered by persons fluent in the workers’ native languages (e.g., employees of manpower agencies or immigrants’ spouses), which is noticeably different from the OSH training programs for native workers. Because blue-collar foreign workers in Taiwan receive almost all workplace safety and health information through translators, whether or not those workers understand the workplace safety and health rules depends on the translators assisting the instructors. Unfortunately, Taiwan’s lack of regulations concerning the qualifications of translators in blue-collar foreign workers’ OSH training programs makes it uncertain whether the translators can correctly convey the professional instructions as given by a qualified instructor, whether the blue-collar foreign workers can effectively understand the training content, and whether there is a gap between the instructions given and perceived. Moreover, the language barriers often make blue-collar foreign workers unwilling to raise questions about safety and health problems on job sites during training sessions—hence the difficulty in obtaining workplace safety and health information. To ensure all blue-collar foreign workers in Taiwan receive OSH education in a correct and professional manner, it is advised that a set of qualification criteria be introduced for the translators of such training programs. That way, blue-collar foreign workers will be further assured of workplace safety in Taiwan.

3. Regulate the longer hours of blue-collar foreign workers’ OSH training programs.

According to the Regulations for Occupational Safety and Health Education and Training [11], a newly hired or incumbent worker shall receive vocational training of no fewer than three hours before being assigned a different job. As stated earlier, blue-collar foreign workers in Taiwan currently receive OSH training mostly from qualified instructors in Mandarin, with on-site interpretation or assistance offered by persons fluent in the workers’ native languages (e.g., employees of manpower agencies or immigrant spouses). The time taken up by translation means that foreign blue-collar workers receive fewer than the required three hours of OSH training. Because foreign workers’ unfamiliarity with Taiwanese languages makes them more accident-prone, it is advisable that such workers receive longer hours of OSH training, not fewer hours than native Taiwanese workers.

## 5. Conclusions

This paper explored Taiwanese manpower agencies’ perspectives in order to establish a mechanism of hazard prevention of occupational accidents for blue-collar foreign workers. Although workers of all nationalities are entitled to job equality and are required to have OSH knowledge, blue-collar foreign workers have relatively greater safety risks in the workplace because they have to adapt themselves mentally and physically to life in Taiwan, which is different from their home countries culturally and/or linguistically. The authorities concerned should therefore attach greater importance to blue-collar foreign workers’ OSH education/training and ensure that they are knowledgeable enough about OSH to enjoy safe workplaces. 

After the authorities and the businesses that hire blue-collar foreign workers establish the mechanism proposed in the study, it is imperative for these entities to set up a performance evaluation system and indicators to assess the hazard prevention mechanism, in order to increase work performance and continually improve the process. Future studies may use the proposed mechanism to set up a performance system and indicators to evaluate the performance of OSH education/training programs for blue-collar foreign workers. Also, though based on the manpower agencies’ perspectives, communication and OSH training/education are the main concerns for the prevention of occupational hazards, future study may consider some other aspects of occupational hazards, such as assignment to more dangerous work or industries and lack of empowerment to refuse dangerous work, to determine whether these aspects cause higher risks of occupational hazards.

## Figures and Tables

**Table 1:** Backgrounds of participants.

No.	Gender	Education	Experience	Contract Factories	Number of Blue-Color Workers with Whom They Have Dealt
1	male	bachelor	5 years 2 months	1	50
2	female	bachelor	5 years 11 months	1	300
Focused interviewers in Taipei					
4	male	bachelor	3 years 2 months	3	250
5	Female	bachelor	5 years 3 months	2	242
6	Female	master	6 years 2 months	2	330
7	Male	bachelor	4 years 5 months	3	285
8	Male	bachelor	5 years 6 months	1	202
Focused interviewers in Taichung					
9	Female	bachelor	2 years 11 months	2	240
10	Female	bachelor	5 years 10 months	2	220
11	Female	master	4 years 6 months	4	332
12	male	bachelor	5 years 4 months	3	235
Focused interviews in Kaohsiung					
13	Male	bachelor	8 years 3 months	3	240
14	Male	Junior college	5 years 7 months	2	250
15	Female	bachelor	3 years 4 months	3	310
16	Female	bachelor	5 years 9 months	3	300
17	Female	Junior college	4 years 8 months	2	250

**Table 2:** Theoretical sampling as compared to comparative and open coding.

Categories (Comparative and Open Coding)	Codes Extracted from the Transcripts
Assistance in selecting blue-collar foreign workers	The nature of job openings at the hiring business (A-1); Selecting the right blue-collar foreign workers (A-2); Seeking the help of foreign-based staffing/placement firms in selecting workers overseas (A-3); Some workers selected/recommended by foreign-based staffing/placement firms are unqualified (A-4); Taiwanese manpower agencies should control the quality of imported human resources during the interviews (A-5).
Helping blue-collar foreign workers communicate with Taiwanese people and adapt to life in Taiwan	Most of the hiring businesses have no more Chinese-language requirements for blue-collar foreign workers than the basic communication skills (B-1); Workers are interviewed in English by manpower agencies in their home countries (B-2); Language barriers are common among business that hire blue-collar foreign workers (B-3).
Preparing the OSH education/training information	In their home countries, most blue-collar foreign workers have received training from manpower agencies in occupation skills, but not OSH knowledge (C-1); It is possible that some blue-collar foreign workers did not receive any training (C-2); Before recruiting, the manpower agency should discuss with the hiring business the potential occupational accidents linked to the job openings, their causes, and other important information (C-3).
Sources of OSH teaching materials	The newly recruited blue-collar foreign workers should receive OSH education/training immediately when they start working for a Taiwanese business (D-1); OSH education/training materials could come from the hiring business, the cabinet-level Council of Labor Affairs (CLA), or information obtained by manpower agencies (D-2); Outdated information in OSH education/training materials (D-3).
In-plant services	New recruits are given access to senior workers’ guidance at the hiring business (E-1); The manpower agencies offer round-the-clock, in-plant services (E-2); Workers receive on-site instructions according to the nature of their tasks (E-3); Only through on-site testing can the employer ensure a blue-collar foreign worker really knows how to perform tasks correctly (E-4); The interpreter’s role as an assistant/adviser for blue-collar foreign workers (E-5); With the help of interpreters, a business may add bilingual warning signs to the machinery and thus protect blue-collar foreign workers (E-6).
Interpreting services as part of the OSH training programs	The interpreters are seriously lacking in OSH-related expertise (F-1); Most interpreters do nothing more than literally translating what the instructor says into English (F-2); The interpreters’ unfamiliarity with OSH instructions may cause biased communication and send the wrong message (F-3); Most businesses that hire blue-collar foreign workers give OSH training through oral instructions (F-4); Blue-collar foreign workers do not have a realistic sense of potential occupational accidents (F-5); Images/photos of real disaster scenes make it easier for blue-collar foreign workers to grasp the consequences of occupational accidents (F-6); The interpreters have poor comprehension of OSH instructions and may not understand how language usages differ among the workers’ home countries (F-7); Translating instructions on the sole basis of the Taiwanese employers’ terminology may cause misunderstandings, rather than communicating the message to blue-collar foreign workers (F-8); The language skills vary among blue-collar foreign workers (F-9).
Giving advice to blue-collar foreign workers on the abilities required for life in Taiwan	Helping blue-collar foreign workers adapt to their Taiwanese jobs (G-1); The blue-collar foreign workers’ adaptability to the overall living environment and their jobs in Taiwan (G-2); Working in an unknown environment (G-3); Avoid misunderstandings due to poor communication (G-4) and/or cultural differences; Manpower agencies should keep track of blue-collar foreign workers after they arrive in Taiwan to ensure their adaptability to the new environment (G-5); Ensuring the blue-collar foreign workers’ occupational safety (G-6); Relying on the government for effective supervision (G-7).

**Table 3:** Comparative and open coding as compared to selective coding.

Concepts (Selective Coding)	Categories (Comparative and Open Coding)
An intermediary institution that selects blue-collar foreign workers for the employers	Assistance in selecting blue-collar foreign workers
A bridge of communication between blue-collar foreign workers and employers	Helping blue-collar foreign workers communicate with Taiwanese people and adapt to life in Taiwan
Gathering information for OSH education/training	Preparing OSH education/training information
Assistance in OSH education/training for blue-collar foreign workers	Sources of OSH teaching materials
	In-plant services
	Interpreting services as part of the OSH training program
Helping blue-collar foreign workers adapt to life in Taiwan	Giving advice to blue-collar foreign workers on the abilities required for life in Taiwan
